# A Narrative Review on the Update in the Prevalence of Infantile Colic, Regurgitation, and Constipation in Young Children: Implications of the ROME IV Criteria

**DOI:** 10.3389/fped.2021.778747

**Published:** 2022-01-05

**Authors:** Leilani Muhardi, Marion M. Aw, Mohammed Hasosah, Ruey Terng Ng, Sze Yee Chong, Badriul Hegar, Erick Toro-Monjaraz, Andy Darma, Merih Cetinkaya, Chung Mo Chow, Urszula Kudla, Yvan Vandenplas

**Affiliations:** ^1^Friesland Campina AMEA, Singapore, Singapore; ^2^Department of Paediatrics, Khoo Teck Puat-National University Children's Medical Institute, National University Health System, Singapore, Singapore; ^3^Department of Paediatrics, Yong Loo Lin School of Medicine, National University of Singapore, Singapore, Singapore; ^4^Department of Pediatric, King Saud Bin Abdulaziz University for Health Sciences, Jeddah, Saudi Arabia; ^5^King Abdullah International Medical Research Center (KAIMRC), Jeddah, Saudi Arabia; ^6^Department of Pediatrics, University of Malaya, Kuala Lumpur, Malaysia; ^7^Department of Pediatrics, Hospital Raja Permaisuri Bainun, Ipoh, Malaysia; ^8^Department of Pediatrics, Faculty of Medicine, Universitas Indonesia, Jakarta, Indonesia; ^9^Unit of Physiology and Gastrointestinal Motility, Department of Gastroenterology and Nutrition, National Institute of Pediatrics, Mexico City, Mexico; ^10^Department of Pediatrics, Faculty of Medicine, Universitas Airlangga, Surabaya, Indonesia; ^11^Department of Neonatology, Health Sciences University, Basaksehir Cam and Sakura City Hospital, Istanbul, Turkey; ^12^Virtus Medical Group, Hong Kong, Hong Kong SAR, China; ^13^Friesland Campina, Amersfoort, Netherlands; ^14^UZ Brussel, KidZ Health Castle, Vrije Universiteit Brussel (VUB), Brussels, Belgium

**Keywords:** prevalence, colic, regurgitation, constipation, young children, review

## Abstract

Regurgitation, colic, and constipation are frequently reported Functional Gastrointestinal Disorders (FGIDs) in the first few years of life. In 2016, the diagnostic criteria for FGIDs were changed from ROME III to ROME IV. This review assesses the prevalence of the most frequent FGIDs (colic, regurgitation and constipation) among children aged 0–5 years after the introduction of the later criteria. Articles published from January 1, 2016 to May 1, 2021 were retrieved from PubMed and Google Scholar using relevant keywords. A total of 12 articles were further analyzed based on the inclusion and exclusion criteria. This review consists of two studies (17%) from the Middle East, three (25%) from Asia, two (17%) from the USA, three (25%) from Europe, and one (8%) from Africa. Three studies (25%) were based on data obtained from healthcare professionals, while the rest were parent or caregiver reports. About half of the retrieved studies used the ROME IV criteria. Among infants aged 0–6 months, the reported prevalence of colic ranged between 10–15%, whilst that of regurgitation was 33.9%, and constipation was 1.5%. Among infants aged 0–12 months, the reported prevalence of regurgitation and constipation were 3.4–25.9% and 1.3–17.7%, respectively. The reported prevalence of constipation was 1.3–26% among children aged 13–48 months and 13% among children aged 4–18 years. Despite the large variations due to differences in diagnostic criteria, study respondents and age group, the prevalence of infantile colic was higher, while that for infantile regurgitation and constipation were similar using the ROME IV or III criteria.

## Introduction

The gastrointestinal (GI) tract is a complex organ that plays a primary role in digestion, nutrient absorption, and excretion of waste products (1). In addition, it also has major neural and endocrine functions and is the largest immune organ that is exposed to multiple antigens. It is also home to trillions of microorganisms, including bacteria, viruses, protozoa, and fungi, which together constitute the gut microbiota (2).

The GI tract starts to develop rapidly from 16 days post-conception and continues to mature during the postpartum period (1). It takes time for this digestive system to become fully functional. For example, the production of lipases and bile salts is low in newborn term infants, and the production of enzymes to digest starch and complex carbohydrates reach its optimal level only a half year later (1). Therefore, infants have a relatively immature GI function, especially in the first few months of life, which makes them prone to a variety of Functional Gastrointestinal Disorders (FGIDs) (3, 4).

Regurgitation, colic, and constipation have been reported to be the most frequent FGIDs in the early years (3, 5). Children with a history of FGIDs during infancy were reported to have a higher risk of persistent FGIDs symptoms later in life (6, 7).

The prevalence of functional constipation varies across countries. The overall prevalence of FGIDs among children aged 0–18 years ranges from 7 to 30% (3, 5, 8). The latest studies reported an estimated prevalence of 31.4% among infants aged 0–1 year coming to pediatric clinics in 10 African countries (9), 7% among children aged 0–4 years in China (10) and 22% among children aged 0–5 years in Saudi Arabia (11). One of the reasons for the differences in the prevalence of FGIDs is the use of different diagnostic criteria. The ROME IV criteria for colic were developed mainly by modifying the Wessel's criteria, and the ROME IV criteria for constipation were developed by categorizing children according to toilet-training status (Table 1) (5, 12). This review aims to assess the prevalence of the most frequent FGIDs in children aged 0–5 years after the introduction of the ROME IV criteria in 2016.

**Table 1:** The ROME III and ROME IV criteria for the diagnosis of colic, regurgitation, and constipation.

	**ROME III (12)**	**ROME IV (5)**
Infant colic	Diagnostic criteria must include all of the following in infants from birth to 4 month of age:	Diagnostic criteria must include all of the following in infants from birth to 5 month of age when symptoms start and stop
	Paroxysms of irritability, fussing or crying that starts and stops without obvious cause	Recurrent and prolonged periods of fussing/crying/irritability which cannot be prevented or resolved by care-givers
	Episodes lasting 3 or more hours/day	-
	Occurring at least 3 day/week for At least 1 week	-
	No failure to thrive	No failure to thrive/fever/illness
Infant regurgitation	Diagnostic criteria must include both of the following in otherwise-healthy infants 3 week to 12 month of age:	Diagnostic criteria must include both of the following in otherwise-healthy infants 3 week to 12 month of age:
	Regurgitation 2 or more times per day for 3 or more weeks	Regurgitation 2 or more times per day for 3 or more weeks
	No retching, hematemesis, aspiration, apnoea, failure to thrive, feeding, or swallowing difficulties, or abnormal posturing	No retching, hematemesis, aspiration, apnoea, failure to thrive, feeding, or swallowing difficulties or abnormal posturing
Infant functional constipation	Diagnostic criteria must include one month of at least 2 of the following in infants up to 4 years of age:	Diagnostic criteria must include one month of at least 2 of the following in infants up to 4 years of age:
	Two or fewer defecations per week	Two or fewer defecations per week
	Manual manoeuvres to facilitate in defecations	History of excessive stool retention
	Straining	History of painful or hard bowel movements
	Lumpy/hard stools	History of large diameter stools
	Sensation of anorectal blockage/obstruction	Presence of a large faecal mass in the rectum
	Sensation of incomplete evacuation	
		*For toilet-trained children:*
		At least one episode per week of incontinence after the acquisition of toileting skills
		History of large diameter stools which may obstruct the toilet

## Methods

### Search Strategy

PubMed and Google Scholar were searched to retrieve relevant articles published from January 1, 2016 to May 1, 2021 using the following keywords: prevalence, infant, (young) children, colic, crying/fussing/distress, constipation, gastroesophageal reflux, gastroesophageal reflux disease, and infant regurgitation.

### Study Selection

Inclusion criteria were observational studies with a precise definition of the symptoms of FGIDs (ROME III or ROME IV or stricter), published in English, time of publication: January 2016 to May 1, 2021. Exclusion criteria were intervention studies, the study population was children older than 5 years, systematic reviews, and non-English publications. Relevant publications from the list of references were retrieved and further analyzed to check if they met the inclusion/exclusion criteria. Data retrieval and extraction were performed by one of the authors. The quality of the retrieved articles was not assessed.

## Results

### Study Results and Characteristics

**Figure 1:**
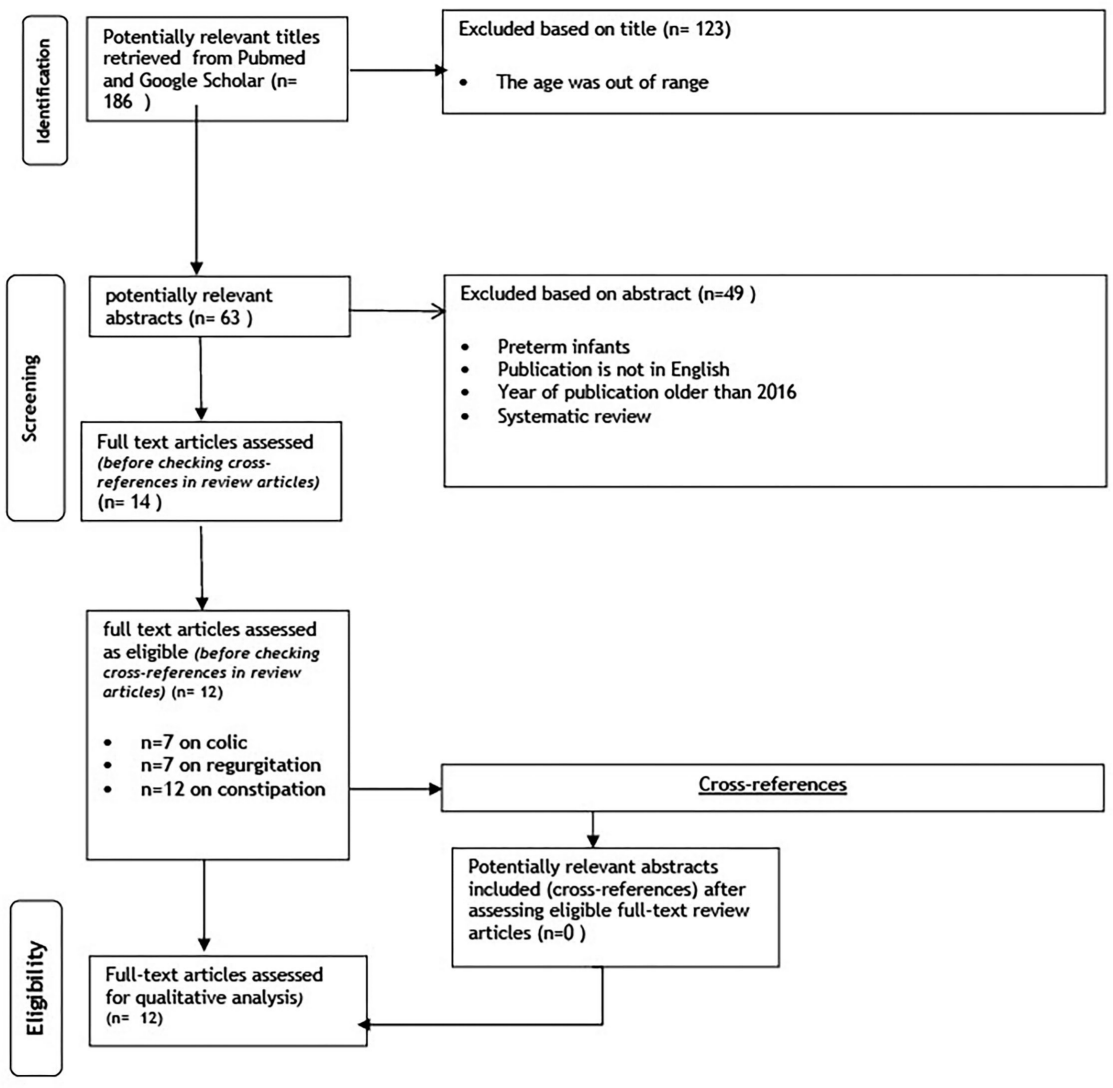
PRISMA diagram flow chart.

The search strategy retrieved 186 articles. Among them, 12 articles fulfilled the eligibility criteria and were included for further analysis (Figure 1). Only three studies (25%) were based on data obtained from healthcare professionals or well-baby clinics (9, 13, 14). Other studies collected data through surveys among caregivers (mostly mothers). There were two studies (16%) from the Middle East (11, 15), three (25%) from Asia (10, 13, 16), two (16%) from the USA (14, 17), three (25 %) from Europe and one (8%) from Africa (9).

### Diagnostic Criteria

Among the seven studies reporting the prevalence of colic, two (28%) used the ROME III criteria (4, 14), four (57%) used the ROME IV criteria (9, 10, 13, 18), and one (14%) applied both the ROME III and ROME IV criteria (17) (Table 2). Among the seven studies reporting the prevalence of regurgitation, one (14%) used the Infant Gastro-Esophageal Reflux Questionnaire Revised (I-GERQ-R) (19), two (28%) used the ROME III criteria (4, 14) and three (42%) used the ROME IV criteria (10, 13, 18) and one (14%) used both the ROME III and IV criteria (17). Among the twelve studies reporting the prevalence of constipation, six (50%) used the ROME III criteria (4, 11, 14–16, 20), five (42%) applied the ROME IV criteria (9, 10, 13, 18), and one (8%) used both the ROME III and ROME IV criteria (17).

**Table 2:** An overview of the retrieved literature.

	**Colic** **(*n* = 7)**	**Regurgitation** **(*n* = 7)**	**Constipation** **(*n* = 12)**
ROME III	2	2	7
ROME IV	4	4	4
ROME III+IV	1	-	1
I-GERQ-R	-	1	-

### Prevalence of Colic, Regurgitation, and Constipation

Despite the variation in the retrieved data in this review, the reported prevalence of colic assessed using the ROME IV criteria was higher than those assessed using the ROME III criteria, whereas the reported prevalence of constipation and regurgitation assessed using the ROME IV criteria were similar to those assessed using the ROME III criteria (Table 3).

**Table 3:** An overview of the prevalence of colic, regurgitation, and constipation assessed using the ROME III and ROME IV criteria among infants aged 0–12 months.

	**0–6 months**		**0–12 months**	
**Prevalence (%)**	**ROME III**	**ROME IV**	**ROME III**	**ROME IV**
Colic	10.4	14.9	4.2–5.9	1.9–19.2
Regurgitation	NA	33.9	8–25.9	3.4–24.1
Constipation	NA	1.5	4.7–17.7	1.3–16.1

*NA, not available*.

Only one study (14%) reported the prevalence of colic assessed using the ROME IV criteria among infants aged 0–5 months (10) (Table 4). The rest of the studies (*n* = 6; 86%) included older infants (aged 0–12 months) for assessing the prevalence of colic using the ROME IV criteria.

**Table 4:** The prevalence of colic in infants aged 0–12 months.

**References**	**Country**	**Study design**	**Participants**	**N**°****	**Criteria**	**Age** **(months)**	**Prevalence** **(%)**
Chogle et al. (14)	Colombia	Questionnaire	Community	259	ROME III	1–4	10.4
Huang et al. (10)	China	Questionnaire	Community	2791	ROME IV	0–6	14.9
Steutel et al. (4)	Belgium, Netherlands, Italy	Doctor-diagnosed	GP	1698	ROME III	0–12	4.2
Robin et al. (17)	USA	Questionnaire	Community	58	ROME III	0–12	5.9
Robin et al. (17)	USA	Questionnaire	Community	58	ROME IV	0–12	5.2
Bellaiche et al. (9)	10 African countries^*^	Questionnaire	Ped	759	ROME IV	0–12	14.9^**^
Chew et al. (13)	Malaysia	Questionnaire	GP	534	ROME IV	1–12	1.9
Beser et al. (18)	Turkey	Doctor's diagnosis	Ped,GE	2383	ROME IV	1–12	19.2

*N°, number of infants included; NA, not available; GP, general practitioner/well-baby clinics; Ped, pediatricians; GE, gastroenterologists; Community = parents/caregivers*.

* *Algeria, Morocco, Tunisia, Mauritius, Madagascar, Senegal Gabon, Congo, Ivory Coast, Cameroon*;

** *combined prevalence of regurgitation and colic*.

The prevalence of colic assessed using the ROME IV criteria was 14.9% (10), whereas the prevalence assessed using the ROME III criteria was 10.4% (14). In studies among infants aged 0–12 months, the reported prevalence of colic assessed using the ROME IV criteria ranged from 1.9 to 19.2% (13, 18).

Two surveys reported that the prevalence regurgitation among infants aged 0–6 months ranged from 34 to 40% (Table 5) (10, 19). These two studies reported conflicting prevalence of regurgitation (3% in China and 18% in France) among infants aged 6–12 months using different criteria (10, 19). One study from the USA reported a similar prevalence of regurgitation of 25% using either the ROME III or ROME IV criteria in the same population of children aged 0–12 months (17). Among children aged 0–12 months, three studies using the ROME III criteria reported a wider range of prevalence of regurgitation (4, 14, 17) than that reported in two studies using the ROME IV criteria (13, 18) (8–26% vs. 10–24%, respectively).

**Table 5:** The prevalence of regurgitation among children aged 0–12 months.

**References**	**Country**	**Study design**	**Participants**	**N**°****	**Criteria**	**Age** **(months)**	**Prevalence** **(%)**
Huang et al. (10)	China	Questionnaire	Community	2791	ROME IV	0–6	33.9
Curien-chotard and Jantchou (19)	France	Questionnaire	Parents	157	I-GERQ-R	6	40
Curien-chotard and Jantchou (19)	France	Questionnaire	Parents	157	I-GERQ-R	10	18
Huang et al. (10)	China	Questionnaire	Community	2791	ROME IV	7–12	3.4
Steutel et al. (4)	Belgium, Netherlands, Italy	Doctor-diagnosed	GP	1698	ROME III	0–12	13.8
Robin et al. (17)	USA	Questionnaire	Community	58	ROME III	0–12	25.9
Robin et al. (17)	USA	Questionnaire	Community	58	ROME IV	0–12	24.1
Bellaiche et al. (9)	10 African countries^*^	Questionnaire	Ped	759	ROME IV	0–12	14.9^**^
Chogle et al. (14)	Colombia	Questionnaire	Community	527	ROME III	1–12	8.0
Chew et al. (13)	Malaysia	Questionnaire	GP	534	ROME IV	1–12	10.5
Beser et al. (18)	Turkey	Doctor's diagnosis	Ped, GE	2383	ROME IV	1–12	13.4

*N°, number of infants included; NA, not available; GP, general practitioner/well-baby clinics; Ped, pediatricians; GE, gastroenterologists; I-GERQ-R, Infant Gastro-eosphageal reflux questionnaire Revised; Community = parents/caregivers*.

* *Algeria, Morocco, Tunisia, Mauritius, Madagascar, Senegal Gabon, Congo, Ivory Coast, Cameroon*;

** *combined prevalence of regurgitation and colic*.

One study reported a similar prevalence of 1.5% for constipation among infants aged 0–6 months and older infants aged 6–12 months (10) (Table 6). Using the ROME III criteria, one study reported a prevalence of constipation of 3% among infants aged 0–12 months (4), whereas one study in Colombia (14) and one study in Turkey reported a prevalence of 16–18% (20). Using the ROME IV criteria, one study that were based on data obtained from parents/caregivers reported a prevalence of 10–12% among infants aged 0–12 months (17), whereas another study that was based on data obtained from pediatricians reported a lower prevalence of 1.3% (13).

**Table 6:** The prevalence of constipation among children aged 0–60 months.

**Author, study year**	**Country**	**Study design**	**Participants**	**N**°****	**Criteria**	**Age** **(months)**	**Prevalence** **(%)**
Huang et al. (10)	China	Questionnaire	Community	2791	ROME IV	0–6	1.5
Huang et al. (10)	China	Questionnaire	Community	2791	ROME IV	7–12	1.5
Steutel et al. (4)	Belgium, Netherlands, Italy	Doctor-diagnosed	GP	1698	ROME III	0–12	3.0
Robin et al. (17)	USA	Questionnaire	Community	58	ROME III	0–12	4.7
Hisar and Hisar (20)	Turkey	Doctor's diagnosed	Community	203	ROME III	0–12	17.7
Bellaiche et al. (9)	10 African countries^*^	Questionnaire	Ped	759	ROME IV	0–12	10^**^
Robin et al. (17)	USA	Questionnaire	Community	58	ROME IV	0–12	12.1
Chew et al. (13)	Malaysia	Questionnaire	GP	534	ROME IV	1–12	1.3
Beser et al. (18)	Turkey	Doctor's diagnosis	Ped, GE	2383	ROME IV	1–12	4.6
Chogle et al. (14)	Colombia	Questionnaire	Community	527	ROME III	1–12	16.1
Robin et al. (17)	USA	Questionnaire	Community	238	ROME III	12–36	9.4
Robin et al. (17)	USA	Questionnaire	Community	238	ROME IV	12–36	18.5
Huang et al. (10)	China	Questionnaire	Community	2791	ROME IV	12–48	7.0
Steutel et al. (4)	Belgium, Netherlands, Italy	Doctor-diagnosed	GP	1053	ROME III	13–48	9.7
Chogle et al. (14)	Colombia	Questionnaire	Community	656	ROME III	13–48	26.8
Al Ghamdi and Alfetni (11)	Saudi Arabia	Questionnaire	Community	80	ROME III	0–60	22.5
Park et al. (16)	Korea	Questionnaire	Community	217	ROME III	25–84	8.5
Altamimi et al. (15)	Jordania	Questionnaire	Community	815	ROME III	4–10^*^^#^	13.4
Robin et al. (17)	USA	Questionnaire	Community	959	ROME III	4–18^*^^#^	12.9
Robin et al. (17)	USA	Questionnaire	Community	959	ROME IV	4–18^*^^#^	14.1

*N°, number of infants included; NA, not available; GP, general practitioner/well-baby clinics; Ped, pediatricians; GE, gastroenterologists; I-GERQ-R, Infant Gastroe-eosphageal reflux questionnaire Revised; Community=parents/caregivers*.

* *Algeria, Morocco, Tunisia, Mauritius, Madagascar, Senegal Gabon, Congo, Ivory Coast, Cameroon*;

* *combined prevalence of constipation and colic*.

# *Age in years*.

Using the ROME III criteria to diagnose constipation among children aged 13–48 months, one study reported a prevalence of 10% in Belgium, Italy, and the Netherlands (4) and 27% in Colombia (14). Two different surveys conducted at different points in time in Turkey reported different prevalence of constipation among infants aged 1–11 months; a survey among parents reported a prevalence of 17.7% assessed using the ROME III criteria (20) whereas the other reported a prevalence of 4.6% assessed based on physician diagnoses using the ROME IV criteria (18). One study from the USA reported a similar prevalence of constipation (13%) using either the ROME III or ROME IV criteria in the same population of children aged 4–18 years (17).

## Discussion

This narrative review provides an updated estimate of the prevalence of FGIDs in various countries worldwide. There are limited studies regarding FGIDs in infants/young children from Australia Oceania and Latin America. Only a few studies have been conducted in Asia and Africa although these two continents have a large population of children (21).

In addition, most of the retrieved studies were based on parent-reported questionnaires, which could lead to an overestimation of the prevalence of FGIDs compared to diagnosis by healthcare professionals (22). It is expected that depending on health-seeking behaviors in specific region, parents or caregivers may attempt to self-medicate or manage illness at home and seek to consult a physician only when symptoms persist (23, 24). Although, there are limited studies comparing parents/physician diagnosis on the prevalence of FGIDs, the differences between perception of the symptoms between parents and physicians' have been widely reported and recognized for other clinical conditions. For example, the definition of wheezing is reflected in the differences seen through estimating the prevalence of asthma (25, 26). These differences could also explain a lower prevalence of FGIDs consistently reported by healthcare professionals compared to that reported by parents (13, 18).

Although about half of the retrieved studies used the ROME IV criteria (Table 2), the data obtained in this review largely varied due to differences across studies such as the diagnostic criteria, study respondents, age group, and geographical location. For example, one study that was based on data obtained from similar types of respondents reported an almost similar prevalence of colic and regurgitation using either the ROME III or ROME IV criteria in similar types of study populations (17). However, the prevalence of constipation in this cohort assessed using the ROME IV criteria was twice as high as that assessed using the ROME III criteria. This is quite difficult to explain. Whilst the criteria *per se* for defining constipation has not changed from Rome III to Rome IV, Rome IV is more explicit in defining constipation in toilet-trained children (27). Another example was from the two surveys on prevalence of constipation conducted in Turkey. These surveys used different diagnostic criteria and included different study populations with different types of respondents; thus they reported different prevalence rates (18, 20). In addition, this review has several limitations such as the use of only two databases and a lack of assessment of the validity and quality of each retrieved study.

Several studies in this review reported the prevalence of colic in infants older than 6 months; despite, the experts' consensus that infantile colic should resolve within the first half year of life (5). The reported prevalence of colic among infants aged 0–5 months assessed using the ROME IV criteria was higher than that assessed using the ROME III criteria (14.9 vs. 10.4%) (Table 4). The range of the prevalence of colic among infants aged 0–12 months assessed using the ROME IV criteria was also larger than that assessed using the ROME III criteria (1.9–19.2% vs. 4.2–5.9%). This increase in prevalence could be due to the fact that the diagnosis of colic using ROME IV is less defined and applies to a larger age group (up to 5 months) compared to those of ROME III (5). In addition, geographical differences in gut microbiota, mode of delivery, sex, place of residence, breastfeeding exclusivity (10, 28) and maternal smoking (29) could also influence these differences. In the future, an age-specific prevalence at each month in the first 6 months of age could be more clinically relevant than an overall prevalence calculated for the first 5 months of life.

The range of the prevalence of regurgitation among infants aged 0–6 months assessed in this review (34–40%) is similar to that reported in a previous systematic review (23–40%) (30). However, this prevalence is lower than that reported in a study conducted in Indonesia in 2005 (44%) among only infants aged 4–6 months using the Gastroesophageal Reflux Disease (GERD) definition (31). In addition, there seem to be regional differences in the prevalence of regurgitation; the reported prevalence of regurgitation among infants aged 6–12 months in France (19) was ten times higher compared to that in China (10). These differences could be attributed to cultural influences in the perception of symptom severity in addition to genetic or feeding differences. A study aiming to validate cow milk symptom score data in relation to reflux reported that healthy Polish infants cried more than the Italians due to differences in parental tolerance (32).

This review is able to provide an age-specific prevalence for constipation. The overall prevalence of constipation (1.5–17%) among children aged 0–12 months seems to be lower than that reported in two previous systematic reviews (0.7–30% and 0.5–32.2%) (8, 33). However, a systematic review of studies conducted in China reported a similar prevalence of constipation (14.4%) among children younger than 2 years of age using earlier ROME II or ROME III criteria (28).

The prevalence of constipation among children aged 3–18 years in this review (13%) was higher than that reported in a recent publication from the western province of Saudi Arabia using the ROME IV criteria (4.7%) (34). In general, the prevalence of constipation increases with age regardless of the diagnostic criteria used.

Knowledge on updated data on the prevalence of FGIDs in various age groups could further help healthcare professionals in managing these symptoms and in reassuring parents or caregivers (35). Prevalence assessed based on physician-diagnosed FGID symptoms might provide a more accurate estimate. However, the primary data collection might be difficult in the near future as there has been a shift of attention to pediatric health during the COVID-19 pandemic (36). Therefore, a multi-country survey among healthcare professionals could provide a better estimate of the magnitude of FGID symptoms. This approach could also capture the differences in genetics, geographical location, symptom's interpretation/ tolerance. It can also serve as a bridging approach during this prolonged COVID-19 pandemic.

In conclusion, despite the high variability in the retrieved data, the reported prevalence in this review for infantile colic was higher when assessed using the ROME IV criteria then those using the ROME III criteria. The prevalence of infantile regurgitation and constipation were similar using either the ROME IV or III criteria.

## Author Contributions

YV, UK, and LM provided a conceptualization of the writing. LM conducted literature search and analysis. All authors reviewed and edited the drafts, read and agreed to the published version of the manuscript.

## Conflict of Interest

LM and UK were employed by Friesland Campina. The remaining authors declare that the research was conducted in the absence of any commercial or financial relationships that could be construed as a potential conflict of interest.

## Publisher's Note

All claims expressed in this article are solely those of the authors and do not necessarily represent those of their affiliated organizations, or those of the publisher, the editors and the reviewers. Any product that may be evaluated in this article, or claim that may be made by its manufacturer, is not guaranteed or endorsed by the publisher.

## Appendix 1

**Table A1:** Search strategy.

“functional gastrointestinal disorder*” OR “FGID*” OR “colic” OR “crying” OR “fussing” OR “distress” OR “gastroesophageal reflux” OR “regurgitat*” OR “GER*” OR “constipation”	AND
“infant*” OR “neonat*” OR “toddler*” OR “child*” OR “pediatric” OR “pediatric” OR “newborn” OR “baby” OR “babies” OR “young children”	AND
January 1, 2016- May 1, 2021	
